# Clonal Neoantigen: Emerging “Mechanism-based” Biomarker of Immunotherapy Response

**DOI:** 10.3390/cancers15235616

**Published:** 2023-11-28

**Authors:** John Nemunaitis, Laura Stanbery, David Willoughby, Ernest Bognar, Scott Brun, Adam Walter, Bradley J. Monk, Rodney P. Rocconi, Khalil Choucair, Robert L. Coleman

**Affiliations:** 1 Gradalis, Inc., Dallas, TX 75225, USA; 2 Frontage Laboratories Inc., Deerfield Beach, FL 33442, USA; 3 Gold Mast Consulting, LLC, The Woodlands, TX 77380, USA; 4 HonorHealth Research Institute, College of Medicine, University of Arizona, Phoenix, AZ 85012, USA; 5 School of Medicine, Creighton University, Phoenix, AZ 85012, USA; 6 University of Mississippi Medical Center, Jackson, MS 39216, USA; 7 Department of Medical Oncology, Barbara Ann Karmanos Cancer Institute, Wayne State University, Detroit, MI 48201, USA; choucairk@karmanos.org; 8 US Oncology Research, The Woodlands, TX 77380, USA

**Keywords:** clonal neoantigen, biomarker, immunotherapy, cancer, Vigil

## Abstract

**Simple Summary:**

Persistent DNA mutations that affect cellular growth and survival result in the transformation of normal cells into malignant cells. These initiating mutations that involve all of the cancer cells are called clonal mutations. Clonal mutations expressed on the surface provide a distinguishable target for immune cells to identify and grab the cancer. Immune effector cells released to attack cancer by checkpoint inhibitor therapy do not kill the cancer unless they can recognize it. Retrospective data has shown improved survival in patients who receive checkpoint inhibitors and have a high clonal neoantigen profile compared to those who do not. Vigil^®^ is a novel immune technology that is designed to increase clonal neoantigen targeting immune effector cells. Recurrence-free survival and overall survival advantage vs. control has been observed with Vigil^®^ in a randomized trial. Vigil’s ability to increase clonal neoantigen targeting effector cells may be a critical link to achieving more consistent immunotherapy activity.

**Abstract:**

Clonal mutations represent the initiating molecular defects related to cellular transition of a normal phenotype to a malignant phenotype. Molecular genomic assessment utilizing next generation and whole exome sequencing is now being increasingly applied to biomarker determination to refine the use of targeted immune therapies. Case examples followed by retrospective study assessment have convincingly demonstrated clonal neoantigens provide a relevant predictor of response to checkpoint inhibition. A meta-analysis, by Litchfield et al., of over 1000 cancer patients from 12 landmark trials demonstrated no clinical benefit to checkpoint inhibitor (CPI) therapy in correlation to high subclonal tumor mutational burden (TMB), whereas high clonal TMB was found to be significantly correlated with better overall survival (*p* = 0.000000029). We discuss the mechanism of clonal vs. subclonal neoantigen targeting relationship to homologous recombination proficient (HRP) profile, evidence of preclinical and clinical benefit related to clonal neoantigens, and review a novel developing therapy called Vigil^®^, designed to expand the clonal neoantigen targeting effector cell populations. Vigil^®^ is an autologous cellular immunotherapy which is designed to carry the full set of personal clonal neoantigens. Phase 2b results demonstrate a durable recurrence-free survival (RFS) and overall survival (OS) advantage for Vigil^®^ in a subset ovarian cancer population with an HRP cancer profile.

## 1. Introduction

In order for immunotherapy to be effective in shrinking a tumor, the immune system effector cells must be able to recognize the tumor cell as foreign. Cancer cells produce factors that cripple recognition and induce immune suppression. Recognition of cancer as a foreign entity is critical in generating a meaningful immune-induced anti-cancer response. This process occurs when tumor cells release protein fragments during necrotic or apoptotic cell death and the protein fragments are taken up by dendritic cells, processed to peptides, and presented on the cell surface as a part of complexes with major histocompatibility complex (MHC) I molecules. CD8+ T cells that recognize non-self antigen via dendritic cell interaction become activated and are then able to recognize tumor cells as foreign. If sufficient this stimulates apoptosis [1]. Neoantigens, which are the immune targets of cancer recognition, ideally will need to be involved and recognized in all cells comprising a tumor. Clonal neoantigens are the neoantigens present in every cancer cell, representing the original driver mutations initiating a malignant phenotype, whereas subclonal neoantigens are present in only subsets of the total tumor population, having arisen later in the evolution of the tumor. Subclonal neoantigens affect the heterogeneity of cancer and may, in some cases, affect the variance in response to treatment. Targeted immunotherapy has been proposed to be most effective when administered earlier in treatment, prior to recurrence and multiple DNA altering chemotherapy courses, which induce subclonal neoantigen accumulation over time. As cancer cells evolve, particularly under therapeutic pressure, resistance signals develop through molecular selection and subpopulations with resistant molecular profiles often prevail [2,3,4,5]. Many examples of induced resistance to clinical interventions have been demonstrated [6,7,8,9]. Tumor DNA repair efficiency regulates the mutation rate. For example, tumors with increased DNA repair deficiency associated with a *BRCA1/2* mutant and/or a homologous recombination-deficient (HRD) molecular profile are associated with higher mutation rates, whereas homologous recombination-proficient (HRP) cancer profiles are associated with stable DNA repair and lower mutation rate. Accordingly, this results in fewer non-clonal neoantigens and less cancer neoantigen noise, which theoretically improves the effectiveness of anti-cancer targeting. In addition, Russo et al. recently demonstrated downregulation of DNA repair machinery as a key driver of mutations associated with increased precision therapeutic pressure-induced resistance in colorectal cancer [9]. Consequently, tumors with intact homologous recombination DNA repair (i.e., HRP subtype) are expected to better preserve clonal neoantigens across all cells comprising the malignancy.

Immune checkpoint inhibitor (CPI) therapy, which requires recognition of tumor neoantigens for effect, has dramatically changed cancer management, demonstrating clinical benefit in multiple solid tumors [10,11,12]. However, in many cancers, CPIs have shown therapeutic efficacy limited to smaller subpopulations. It has been well established that neoantigen specific effector cells (CD8+ T cells) play a major role [13,14,15,16,17] in engaging tumor cells following initiation of CPI therapy [18]. Although many other components of the immune response support CPI activity (CD4+ T cells, NK cells, dendritic cells and myeloid cells) and modulate the CD8+ T cell directed attack [19]. The most effective anti-cancer response to CPI therapy is dependent on presence of neoantigen specific CD8+ T cell targeting effector cells which are able to induce direct anti-cancer activity [20,21] by targeting specific neoantigens. For complete immune system control and elimination of all the cancer cells, there must be targeting of neoantigens contained on all cancer cells, the clonal neoantigens [22,23]. Clonal neoantigen targets have limited to no advantage as a biomarker to chemotherapy or other non-immune targeted therapies. Although clonal mutations demonstrating resistance to targeted therapy may be of novel relevance to subsequent clonal neoantigen determination, work by Wu et al. [24] demonstrated response to CPI (nivolumab) in an advanced metastatic non-small cell lung cancer (NSCLC) patient who demonstrated progressive disease following acquired resistance to epidermal growth factor receptor-tyrosine kinase inhibitor (EGFR-TKI) at the development of an EGFR exon 19 deletion mutation. A T cell response to clonal neoantigens encoded by the EGFR 19 deletion was demonstrated. This patient achieved a durable partial response lasting over a year after treatment initiation with nivolumab. We will discuss the role of clonal neoantigens in the treatment of a targeted immune response, particularly addressing the clonal neoantigen role in the CPI effect. We will also focus on the clinical management of advanced cancer, including novel approaches in clinical development, such as Vigil^®^, that can enhance anti-cancer activity via induction of effector cell responses targeting clonal neoantigens.

## 2. Relevance of Clonal Mutation Assessment

DNA repair pathways are a critical determinant of cancer mutational activity, which lead to neoantigen expression [25]. For cancer to evolve, driver clonal mutations are required, which cause an event within a normal cell to transition to a cancer cell phenotype. These clonal mutations are a small proportion of the total TMB at onset of cancer and most commonly involve signal pathways associated with cancer growth, spread, and survival [26,27]. Generally, cancer growth is initiated in the body many years before discovery, and most such transformed cells are controlled and eliminated by the innate immune response. However, once the minimum cancer survival support pathways are met, subsequent divergent mutational events occur following further cell divisions. These generate subclonal mutations which are observed at later stages of cancer evolution [23,28,29]. However, subclonal mutations are less effective (compared to clonal) at inducing T cell populations with capacity to bind and react to all cancer cells. Subclonal neoantigens are not contained on all cancer cells. Moreover, subclonal neoantigens that present on tumor cell subpopulations are thought to facilitate tumor escape through the outgrowth of antigen-deficient cells leading to an ineffective dendritic response [30,31,32,33].

The immune system’s ability to detect and eliminate clonal neoantigen-bearing cells depends on the clonal neoantigen fraction within the tumor [34], as well as on the quality and quantity of clonal neoantigen targeting effector cells. Attempts to achieve immune control at all metastatic disease sites by targeting subclonal antigens are not effective as the same subclonal neoantigens are generally not contained in all cancer cells at all metastatic sites. Preclinical studies involving a murine model of controlled intratumor heterogeneity, which mixed multiple immune susceptible clones (i.e., of different subclonal neoantigens) actually resulted in the development of a polyclonal, immune-resistant tumor. This work highlights the resilience of cancer-resistant clones [35].

Effective immune therapy involves effector cells that contain “trained” T cell receptor (TCR) regions to bind and react to neoantigens on all cancer cells, not just subpopulations. Measurement of the clonal tumor mutation burden (cTMB) prior to CPI therapy is thus a logical approach to help predict the likelihood of a clinical response. The importance of targeting clonal neoantigen expression was suggested by McGranahan et al. [23] based on work performed to assess the response of melanoma and lung cancer patients to CPI therapy. Most mutation-driven neoantigens are derived from passenger mutations, which are divergent subclonal mutations acquired following clonal driver mutations. Subclonal mutations can actively drive cancer progression via cellular subpopulations that provide a selective advantage supporting further tumor growth and/or spread. Despite attractive reasons for targeting clonal antigens that are derived from functionally important and conserved proteins, there is still evidence of cancer resistance occurrence. For example, in one closely monitored case, following initial regression of seven colorectal cancer metastases with KRAS-G12D-directed adaptive cell therapy, one metastasis recurred many months later. Following resection, this solitary lesion was found to have a unique strategic mutation involving human leukocyte antigen (HLA) haplotype loss. As a result, this subpopulation of cancer cells developed resistance to the KRAS-G12D adaptive cell therapy, revealing a directly related mechanism of tumor immune evasion [36]. In another study, the metastasis of a primary tumor bearing an immunogenic BRAF clonal neoantigen (V599E) induced an HLA-restricted cytotoxic T cell response to the clonal BRAF neoantigen in the metastatic lesion as the inciting event. However, at recurrence there was no trace of the inciting oncogenic target mutation on follow up sequencing analyses [37]. These are examples of subsequent “strategic” mutations enabling immune resistance following targeted therapeutic intervention. Such reports exemplify limits of single agent targeted therapies, even when directed against a clonal neoantigen. Future work should involve a strategy based on combinatorial or sequential approaches which target multiple clonal neoantigens, if not all clonal neoantigens. To address the shortcoming of selecting limited targets, autologous cellular therapy (as opposed to allogenic) with demonstration of broad clonal neoantigen expression is a logical consideration. As previously discussed, increasing the number of expressed subclonal neoantigens hinders optimal cognate peptide–MHC complex affinity [30] and thus limits the effectiveness of the immune response. An increasing proportion of subclonal neoantigens adversely affects immune response to clonal neoantigens, limiting the therapeutic effect [30].

UVB mutagenesis involving syngeneic mice is a routine model for carcinogenesis induction [38] associated with DNA disruption and associated impairment of DNA. Wolf et al. showed that a reduction of subclonal neoantigens correlated with response improvement in conjunction with decreased tumor growth [35]. Similarly, in NSCLC, exposure to tobacco increases the mutational load of tumors, and more precisely, mutations that result in transversions [39,40]. Defects in DNA-repair pathways could also lead to further increases in the number of mutations and subsequent subclonal neoantigens through a hypermutator phenotype [41,42]. Retrospective clinical data also show cancer patients receiving CPIs with high tumor subclonal neoantigen content and associated decrease in overall survival (OS) compared to similar patients with lower subclonal neoantigen content and high clonal neoantigen burden [23,42].

The robust DNA repair function in HRP, a *BRCA1/2*-wild type (BRCA-wt) tumor molecular profile in ovarian cancer patients, has been suggested as a potential mechanism to explain the increased immune response and subsequent survival benefit observed in patients treated with the novel immunotherapy Vigil^®^ [43]. Survival benefit (discussed in detail in the next section) has been shown in this population (BRCA-wt, HRP) compared to similar placebo-treated patients [44,45,46]. Of note, Vigil^®^ supported the generation of specific immune responses to the patient’s own tumor antigens, as evidenced by an ELISpot assay measuring effector cell driven anti-cancer attack. The improved response in BRCA-wt/HRP subtype tumors is hypothesized to be a result of the higher clonal neoantigen mutational burden expected in tumors with preserved DNA repair pathways. Confirmatory work to evaluate clonal neoantigen burden and correlation to Vigil^®^ clinical response is ongoing.

## 3. TCR Interaction and the Role of Clonal Neoantigens

**Figure 1:**
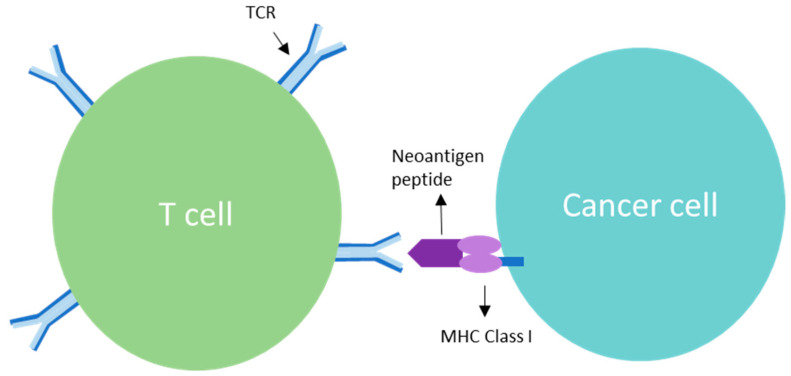
T cell receptor (TCR) is a complex of integrated membrane proteins that participate in activation of T cells in response to clonal neoantigens. Stimulation of TCR is triggered by major histocompatibility complex molecules on tumor cells displaying the clonal neoantigen.

T cells are activated through specific TCR-antigen interactions. V(D)J recombination can generate a massive diversity of T cell clonotypes via thymus interaction followed by positive and negative selection processes, yielding as many as 10^10^ circulating T cell clonotypes [47]. Each clonotype TCR can bind individual T cell antigens, thereby defining T cell specificity. T cell antigens are presented in two types of MHC molecules, termed HLAs. MHC class I (MHC-I) molecules are expressed by all nucleated cells, whereas MHC class II (MHC-II) molecules are expressed by antigen-presenting cells (APCs), epithelial cells and some tumors [48]. TCR binding and relationship to relevant effector cell is shown in Figure 1. Anti-tumor T cell immune response is antigen-specific and antigen selective [49]. Clinical improvement observed with TCR-engineered adoptive cell transfer treatment approaches are obvious examples of the therapeutic power of these activities [36,50]. Lifileucel (LN44), in particular, has demonstrated robust evidence of clinical activity in advanced melanoma including in those who failed CPI therapy [51].

Mutation-derived neoantigens are aberrant proteins derived from cancer-distinct sequences encoded by somatic point mutations, frameshifts or chromosomal abnormalities. Non-synonymous mutated proteins can lead to tumor-specific antigen generation if their intracellular degradation results in neopeptide HLA-binding [52]. These amino acid changes may alter the immunogenicity of an HLA-binding peptide [53] or, if they occur in anchor positions, can turn a non-binding sequence into an HLA-binding one [54]. Additionally, a mutated amino acid could also give rise to a new proteasomal cleavage site, thus allowing peptide processing and HLA loading [55]. Evidence of the role neoantigens play in immune-mediated tumor regression and improved survival is supported by the association between TMB, in particular clonal TMB, and immunotherapy response [22]. However, for response related to neoantigen exposure, T cell infiltration to the tumor environment needs to take place in order to generate a T cell response. T cell priming is achieved through serial binding of multiple MHC-TCR binding complexes. Subclonal neoantigens result in suboptimal T cell priming and activation since the subclonal neoantigens are not located on all cancer cells compared to clonal neoantigens which are located on all cancer cells. Clonal neoantigens in essence result in a more durable T cell response and activation. Thus, T cell binding via TCR region interaction with clonal neoantigens induces a more effective immune effector response and release of cancer cytotoxic cytokines leading to optimal anti-cancer activity.

**Figure 2:**
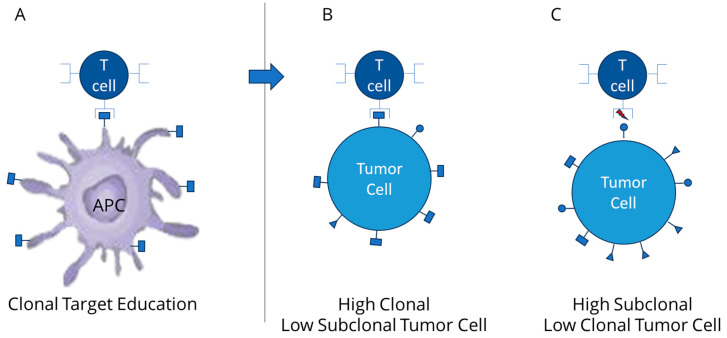
(**A**) expansion of T cell TCR targeting clonal neoantigen via dendritic cell response; (**B**) improved activity of T cell activity against high clonal neoantigens (low subclonal); (**C**) limited T cell activity against high subclonal (low clonal neoantigens).

A T cell response involves three stages of activation to generate anti-cancer activity (Figure 2). First is activation as related to foreign antigen stimulation to which clonal neoantigen provides optimal signal. Stage 2 involves differentiation, which requires complex immune effector cell interaction against the expanded cancer identifying neoantigens. Stage 3 involves a memory cell development and establishes a longer lasting almost “preventative” stage attempting to provide durability to the anti-cancer response [56,57]. It is important to note however, that the T cell infiltration process itself (hot tumor microenvironment) has been characterized as a prognostic indication of good response and has been identified as a biomarker of a CPI response benefit in some patient populations [58,59]. Sufficient infiltration into the tumor microenvironment along with successful activation of effector T lymphocytes against tumor cells has been identified as predictive for response [60,61,62] to immunotherapies such as Vigil [45,46] which is designed to increase clonal neoantigen targeting T effector cells and CPIs which release inhibitory components of cancer protection to enhance access to tumor cells, even those contained within fibrotic matrix.

Optimal TCR peptide–MHC interactions can then take place under conditions that maximize serial engagement of clonal effector cells of a suitable affinity so that signal effectivity and dissociation can readily take place. Minimum affinity is required for productive signaling at each TCR [63,64,65], high affinity interactions may also impair overall T cell activation through prolonged dwell times and reduced serial TCR binding [30,66]. Increased peptide–MHC density has been shown to overcome the impairment of T cell activation by high affinity interactions as the abundance of peptide–MHC complexes compensates for reduced serial TCR engagement due to longer dwell times [31]. Clonal neoantigens, by virtue of being expressed in all tumor cells, appear to provide the requisite peptide–MHC density to engage more TCRs than subclonal neoantigens, thereby overcoming suboptimal binding affinity. As such, clonal neoantigens demonstrate more effective capacity to activate T cells compared to subclonal neoantigens. Engagement of multiple TCRs is required for efficient T cell activation, which further supports the rationale of using autologous tumor tissue, which by definition will contain all available clonal neoantigens, as a source for “TCR training”. Cognate peptide–MHC complexes with optimal affinity (i.e., high enough to signal, low enough to dissociate and engage further TCRs) are able to serially engage TCRs for effective T cell signaling. T cell activation is hindered by sub-optimal cognate peptide–MHC complex affinity. Subclonal neoantigens are especially handicapped by reduced serial TCR engagement due to relatively reduced peptide–MHC density. Increased peptide–MHC density of clonal neoantigens (present on all cancer cells) can overcome the limitations of non-optimal TCR affinity as there is less reliance on serial engagement of TCRs [30]. Clinical evidence related to natural immune surveillance also demonstrates OS and progression-free survival (PFS) of untreated NSCLC can be predicted by quantifying clonal versus subclonal neoantigen concentration or TMB. In addition to evidence supporting clonal neoantigen targeting, some have suggested that persistent mutations that are not clonal may also affect cancer response [67] as these mutations likely occurred early in the tumor evolution (before the copy number gain event).

Several other groups have also studied response to CPI treatment in patients with high clonal TMB or neoantigen levels and have demonstrated improved OS and PFS in comparison to patients with low clonal TMB or high subclonal TMB/neoantigen expression [23,30,42,68,69]. A limit to clonal antigen assessment involves the complexity of the molecular sequencing required combined with the algorithm-based thresholds to determine definition of clonal vs. subclonal populations. Consequently, such assessment is not a routine process of application in current use for clinical management.

## 4. Clonal Neoantigen Identification and Analysis

Several biomarkers have demonstrated the ability to predict response to CPI, including most notably PD-L1 expression, although response cutoff thresholds have varied across approved agents, ranging from 1% to 50% expression levels [70,71]. Other companion diagnostic indicators, such as microsatellite instability (MSI) high assessment and TMB, also have levels of inconsistency with patient response. There are numerous examples of benefit and lack of benefit above and below thresholds set for biomarker predictability. Inconsistency demonstrated may be related to underlying clonal vs. subclonal TMB expression within each subgroup, which was not tracked. Additionally, inconsistency in some response correlation could be related to varied TMB levels depending on malignancy biopsy site [72].

The advent of next-generation sequencing (NGS) has allowed the systematic, unbiased survey of mutations from individual tumors [73]. These data, in turn, have been used to guide novel target antigen discovery [74,75,76,77] either through T cell-based assays or with HLA peptidomics [74,78,79]. However, assay development for routine clonal neoantigen assessment is complex, costly and not yet readily available for routine clinical management. Data generated from whole-exome sequencing-based screening have been used to demonstrate that tumor-infiltrating lymphocyte (TIL) activity against mutation-derived neoantigens actually exists in the majority of cancers, not just in tumor types known to be amenable to immunotherapy (i.e., melanoma) [78,80]. One of the first attempts at using whole exome sequencing data to characterize neoantigens in breast and colon cancer used binding to HLA-A02:01 to predict mutated peptides [73]. Later the technique was optimized with use of patient-specific HLA allele characterization [25]. 

**Figure 3:**
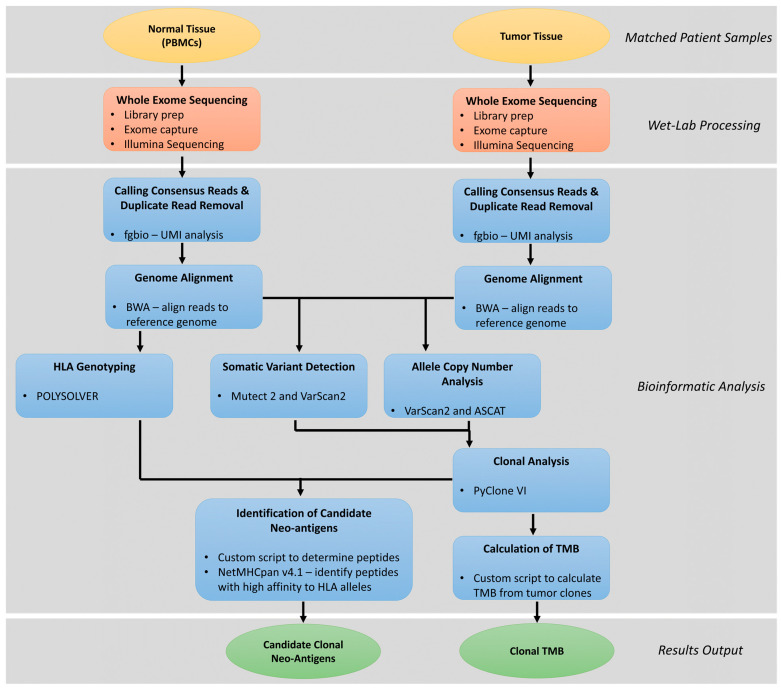
Schematic workflow for identification of candidate clonal neoantigens and determination of clonal tumor mutation burden (TMB).

One approach to the rapid identification of candidate clonal neoantigens, pioneered by McGranahan and coworkers [21], relies on whole exome sequencing of both tumor and normal genomic DNA, and analysis of the resulting sequence data with a multi-step bioinformatic pipeline, that includes somatic variant identification, clonal analysis of somatic variants, HLA typing, and prediction of MHC-I binding affinity for potential neoantigens generated from the sequence of genes harboring non-synonymous variants (Figure 3). Key requirements for success in this process are (1) where possible utilizing multiple tumor samples per patient from different regions of the tumor or metastatic sites, (2) sequencing of the tumor samples at sufficient coverage depth (at least 400–500×) to allow for accurate estimate of the allele fraction of the variants identified with a limit of detection of 5% or lower, (3) making sure the exome capture region provides adequate coverage of the MHC-I and MHC-II loci, and (4) using a library preparation kit for sequencing that incorporated unique molecular identifiers (UMIs) into the libraries. The bioinformatics pipeline begins with the removal of PCR duplicates and correction of sequencing errors based on grouping with the UMIs. Next the deduplicated and error-corrected reads are aligned to the human genome sequence. HLA Class I and II haplotypes are called from the analysis of the aligned and unaligned sequence reads of the genomic DNA from the normal tissue using algorithms such as Polysolver, Optitype, HLA*LA, and HISAT-Genotype [81]. Somatic variants that are predicted to impact the protein coding regions of genes (non-synonymous) are identified by combined analysis of the aligned reads from both the tumor and normal samples using Mutect2 and Varscan2. Likewise, genes or larger chromosomal regions showing changes in copy number in the tumor sample genomic DNA as compared to the paired normal tissue are identified. Somatic variant calls annotated with copy number variation data for each patient are then inputted into PyClone-VI software which uses a Bayesian statistical method to determine clonal populations and associates each variant with one or more specific clones [82]. 

The variants that are associated with a primary clone based on the Pyclone output can be further analyzed to calculate clonal TMB and for the identification of a shortlist of clonal neoantigens. Clonal TMB is calculated as the count of non-synonymous mutations associated with the primary clone per megabase of DNA covered by the exome panel used for sequencing. An initial broad set of candidate clonal neoantigens is generated computationally as the set of all possible 9–11-mer amino acid sequences that contain one or more mutated amino acids that are predicted to arise from one of the primary clonal variants in the DNA. The list of candidate neoantigens peptides for each patient is narrowed by using NetMHCpan and NetMHCIIpan version 4.1 software to predict the affinity of binding of the peptides to the HLA-I and HLA-II molecules, respectively, using the predicted HLA haplotypes and list of candidate peptides as input [83]. Peptides with a predicted IC50 of <500 nM for binding to the HLA molecule presentation sites are retained for further analysis. The candidate clonal neoantigen list can be further narrowed based on examination of the predicted cleavage sites and transport binding sites in the peptide or parent protein sequence. The narrowed candidate list of peptides can then be tested to determine the likelihood that each has been previously presented and encountered by T cells in the patient. This can be accomplished by culturing the patient’s PBMCs in the presence of candidate neoantigen peptides and wild-type sequence controls with periodic replenishment with a media containing IL-2, IL-7, and IL-15, and then measuring the level of T cell activation using an IFN-y ELISpot assay [24].

Immunopeptidomics can also be utilized to determine clonal neoantigens. In this approach, tumor cells are lysed and immunoprecipitation is used to identify MHC-I ligands. These peptides are then analyzed by LC-MS/MS [84]. Numerous examples of clonal and subclonal determination in preclinical testing and retrospective clinical assessment in comparison to clinical benefit parameters are discussed. Establishment of prospective studies to validate relationship of tumor clonal neoantigen or clonal TMB level to clinical benefit derived from immunotherapy treatments are thus justified.

## 5. Clinical Response to CPI Is Variable

Prior to the advent of targeted immune therapy, led by the development of checkpoint inhibitors (CTL-4, PD-1, PD-L1 inhibitors), precision therapy or driver gene-targeted therapies were investigated. Precision therapy is defined as the selection of a specific molecular therapy for management of cancer treatment based on the molecular understanding of the disease. In simple terms, it is a “therapy that targets the etiologic cancer target” which likely involves clonally mutated genes. Such molecular-based targeting requires understanding the role of the signal to driver gene function that generates cancer cell survival advantage. Knowing the gatekeeping function in cancer survival thus provides a justification for therapeutically disrupting this signal. Early signal targets involve the hallmark pathways of cancer survival (i.e., HER2, EGFR, mTOR, ALK, BRAF, RAS, CDK4/6, BRCA1, NTRK) with aggressive expansion of several hundred more now in active preclinical and clinical investigation or already registered as approved products [26].

Precision therapy products have demonstrated significant clinical benefit as measured by OS, RFS, PFS and duration of response [85,86,87,88,89,90]. Such benefit has led to lower hospitalization rates and emergency room visits. Moreover, when precision therapy is utilized early in cancer management, fewer treatment-related toxic deaths and higher payer cost advantage compared to standard chemotherapy are commonly observed [91,92,93]. As such, widespread clinical experience now justifies the implementation of comprehensive molecular profiling assessment early in the diagnosis of a patient’s disease to allow for proper selection from the array of available targeted therapies [94,95]. Benefit related to “targeting the correct target” is an important point in understanding precision immunotherapy (i.e., CPIs) particularly as our awareness of the role of clonal neoantigens expands. The lower toxicity profile of precision therapy also further justifies the utilization of combination treatment with other signal pathway precision therapeutics and/or with precision immunotherapy combinations. Moreover, improved clinical benefits involving PFS and OS have been demonstrated with combination precision therapy as opposed to single agent [96]. Improved clinical benefit has also been suggested with combination immunotherapy (i.e., CTL-4/PD-1/PD-L1 inhibitor combination).

Ipilimumab is the first, and only Food and Drug Administration (FDA) approved CTLA-4 inhibitor, which was initially approved in 2011 based on the pivotal Phase 3 MDX010-020 trial that demonstrated an OS advantage in patients with unresectable, previously treated, late-stage melanoma [97]. Molecular biomarker related advantage was shown in subsequent studies. In CheckMate-069, ipilimumab in combination with nivolumab in BRAF wildtype untreated melanoma, as well as unresectable/metastatic melanoma patients, provided PFS advantage compared to ipilimumab alone (median PFS: not reached vs. 4.4 months; HR: 0.40; 95% CI: 0.23–0.68; *p* < 0.001), granting the combination FDA approval for use in BRAF V600 wild-type tumors [98]. CheckMate-067 trial data provided expanded approval of the combination, regardless of BRAF mutation, with significant OS advantage observed [99]. Others showed that highly soluble PD-1 measured from baseline improved PFS response in patients treated with the combination ipilimumab and nivolumab [100]. Patients with untreated, metastatic colorectal cancer with microsatellite high/mismatch repair deficient (MSI-high/dMMR) tumors treated with the combination of nivolumab and ipilimumab also demonstrated robust and durable clinical benefit with 69% response rate, 84% disease control rate, and 13% complete response (CheckMate-142) [101]. The nivolumab and ipilimumab combination has also shown significant response rates (42% vs. 27%), OS (median not reached vs. 26 months) and PFS advantage (11.6 vs. 8.4 months) compared to standard-of-care sunitinib in treatment-naïve, advanced, intermediate-poor risk clear cell renal cell carcinoma, irrespective of PD-L1 status (CheckMate-214) [102]. 

In 2014, the FDA approved antibodies targeting PD-1 (pembrolizumab and nivolumab), first for the treatment of metastatic melanoma, but as expanded indications were generated, biomarkers of sensitivity continued to vary in threshold with relationship to DNA repair capacity, mutation load and varying levels of PD-L1 expression levels. 

Similarly, avelumab, durvalumab and atezolizumab are all PD-L1-blocking antibodies. Despite a slightly different mechanism of action, they target and disrupt the PD-1/PD-L1 interaction. In a meta-analysis comparing PD-1 and PD-L1 monoclonal antibodies in terms of efficacy and toxicity in NSCLC, there was no difference in response rates (19% and 18.6%) and the overall incidence of adverse events (64% and 66%) between both classes of agents [103]. Table 1 summarizes the major landmark trials, significant responses and indications for each of these CPI agents.

The data discussed above lack a consistent biomarker and threshold, but provide numerous examples of significant benefits of CPI therapy related to varying biomarkers and biomarker thresholds. For example, PFS and OS specifically related to CPI treatment have been shown to be strongly correlated with high TMB in melanoma [135], NSCLC [136] and urothelial carcinoma [131]. However, this is not the case in tumors such as esophageal, gastric, urinary tract or small cell carcinomas. In a study by Rousseau and colleagues, these tumor types were classified as “TMB-insensitive” tumors as their TMB-H status did not correlate with better response to CPI (HR: 0.84; 95% CI: 0.63–1.11), compared to “TMB-sensitive” tumors (NSCLC, melanoma; HR: 0.52; 95% CI: 0.41–0.64) which did correlate with TMB-H [137]. These observations thus warrant exploring deeper into biomarkers of CPI response and support review of the role of clonal TMB or neoantigen expression. 

## 6. Biomarkers of CPI Response

As discussed above, multiple biomarkers have been utilized to define CPI-sensitive populations. There are currently three FDA approved biomarkers of CPI sensitivity: PD-L1 expression, dMMR/MSI-H, and TMB-H. None of these biomarkers are perfectly predictive nor preclude response to CPI. 

PD-L1 is the most extensively studied predictive biomarker of CPI and is typically measured by immunohistochemistry utilizing an array of antibodies. It is quantified in a categorical manner with variable cutoffs of “positivity” (<1 vs. ≥1, <5 vs. ≥5, <10 vs. ≥10, and <50 vs. ≥50). Similarly, differences exist in the source of PD-L1 quantification, whether accounting for PD-L1 on tumor cells only (tumor proportion score- TPS) or its expression on tumor cells, lymphocytes, and macrophages from the tumor microenvironment (CPS: combined positive score) [138,139]. Different FDA approvals for CPI were contingent on PD-L1 status, but at different cut points using various assays. For example, pembrolizumab is approved as monotherapy in metastatic NSCLC patients with PD-L1 >50% (TPS), while investigators in Impassion130, for example, used PD-L1 (TPSS) ≥1% as a cut-off that showed OS benefit to atezolizumab treatment of triple-negative breast cancer [140,141]. On the other hand, Keynote-048 used a CPS score of ≥1% as predictive of benefit from pembrolizumab in patients with head and heck squamous carcinoma [114]. Lastly, although PD-L1 positivity in metastatic NSCLC predicted a higher response rate to pembrolizumab (45%), 15% of PD-L1-negative tumors still responded [142]. Taken altogether, PD-L1 remains an imprecise marker for CPI response due to spatial and temporal heterogeneity within a specific tumor sample and across metastatic sites [143,144], lack of standardization in terms of quantification methods and selection of a threshold to define positivity, and variation between assays and antibody clones [145].

MSI-H/dMMR is the first tissue agnostic biomarker that predicts response to anti-PD-1 in solid tumors in a tissue agnostic manner [146], with high rates of durable response in tumors with somatic dMMR regardless of tissue of origin (ORR: 40% in metastatic colorectal cancer and ORR: 71% in non-colorectal cancers) [41]. Similar supportive data exist for single agent nivolumab and the combination of nivolumab and ipilimumab (ORR: 31% and 55%, respectively) [146,147]. In a pan-cancer study of 27 advanced non-colorectal dMMR tumors, pembrolizumab resulted in an ORR of 34% across tumor types, with a complete response achieved in 10% of patients [148]. 

It has been proposed that MMR/MSI-H Deficient status is a surrogate marker of the presence of neoantigen, defining higher immunogenicity and thus higher response rates to CPI. This, however, is contrary to the predicted expectation projected with clonal TMB or neoantigen display as deficient MMR; MSI-H would be expected to be associated with higher subclonal neoantigens. Subclonal neoantigen signals may still provide immunogenic targets for aggressive subpopulations of cancer growth, possibly associated with a transient, measurable response to CPI therapy. However, as discussed, subclonal neoantigens are not contained in all cancer cells, and typically, with such a target cancer recurrence would be expected to have a limited overall survival impact and possibly a more complete overall response generation in comparison to targeting clonal TMB as a neoantigen. Unlike TMB, which reflects the actual total tumor mutation burden, a deficient mismatch repair status does not precisely predict the level of neoantigen but rather acts as a predisposing mechanism for the accumulation of mutations and neoantigens. Similarly, dMMR/MSI-H status does not distinguish between passenger (subclonal) or clonal antigens.

Early evidence of the predictive value of tumor mutation burden to the outcome to CPI treatment was generated by Snyder et al. by examining a series of outlier responses in melanoma patients who received anti-CTLA-4 therapy [68]. Later, TMB was related to anti-PD-1 therapy [41,42,149,150]. Unfortunately, despite evidence of significance in subset cancer populations, responses have been inconsistent, with evidence of lack of response in subpopulations who fulfill biomarker sensitivity parameters and further evidence of response is occasionally seen in populations fulfilling poor biomarker sensitivity parameters [42,142,151,152,153]. This inconsistency provides difficulties in clinical management decision making. The “sensitive” biomarkers [41,146,148,154,155,156,157] generally apply to about 30% of the cancer population (MSI-H, dMMR, LOH-H, HRD/BRCA-m, TMB ≥10 mu/mb DNA). Tumors with high levels of DNA mismatch repair (MMR) demonstrate a particularly unique subpopulation in terms of sensitivity to CPI treatment. Patients with this molecular profile also tend to have high TMB [41,158,159].

Furthermore, as previously discussed, subclonal TMB appears to not be a significant correlating factor with OS [22,23,42]. The results fairly conclusively support that clonal TMB is an optimal biomarker to predict the OS response to CPI therapy [22]. Thus, clonal TMB appears to be an independent biomarker of improved outcome to clinical response involving CPI treatment that warrants further prospective clinical study assessment. From a biomarker standpoint when assessing the clinical response to CPI therapy, work by Litchfield and others also reveals that reduced or no benefit from CPI therapy is associated with high subclonal TMB [22]. Subclonal neoantigens have in fact been shown to be fairly ineffective at inducing robust induction of activated T cell effector populations capable of demonstrating significant anti-tumor reactivity to whole tumors [22,23,26,30,35,42,68,160]. These results suggest high subclonal TMB is unlikely to provide an optimal target population for CPI therapy and likely would be expected to be a poor target for CAR-T, tumor infiltrating lymphocyte therapy or vaccine approaches.

Another observation supporting the role of clonal TMB emerges from Merkel cell cancer and Hodgkin lymphoma, both tumors with low TMB, but yet with high response rates to CPI. In fact, both malignancies harbor clonal Merkel cell polyomavirus and Epstein–Barr virus genomes [161,162], and these “public” clonal viral neoantigens, when targeted, demonstrate high response rates to CPI therapy [163,164,165] thereby supporting additional evidence that clonal neoantigen density is of greater predictive priority over TMB. Likewise, despite a relatively high TMB, melanomas with higher number of subclonal mutations have been reported to have lower response rates to CPI therapy [35]. The results suggest that clinical management decisions could benefit from routine measurement of clonal neoantigen or TMB. Response to CPI therapy would likely benefit from assessment of clonal and subclonal molecular profiles prior to treatment. This would likely expand the CPI-treated population. Two recent studies involving mice support the evidence that clonal neoantigens are superior at engaging effective tumor immune surveillance. Gejman et al. demonstrated that a minimum fraction of tumor cells must express a neoantigen before adaptive immune rejection, and that there is a dosage threshold which varies between the different neoantigens [34]. In the second study, Wolf et al. showed again in mice that clonal neoantigen tumors were more readily rejected by the immune system compared with heterogenous tumors, independent of total mutational burden [35]. Furthermore, the TRACERx study revealed that tumors with high clonal neoantigen burdens were more densely infiltrated by T cells [166]. 

Many biomarkers used in conjunction with CPI therapy attempt to convey evidence of a “hot” tumor microenvironment, one filled with inflammatory cells, generating evidence of potential for anti-cancer activity. Interestingly, these biomarkers also portray a molecular profile of reduced DNA repair capacity. At least four partially overlapping damage repair pathways operate—nucleotide excision repair, homologous recombination, base excision repair, and end joining. There are numerous hereditary syndromes of defective genome maintenance related to the above mechanisms. All are associated with a predisposition to cancer development (Table 2).

It appears that CPI therapy is less effective in cancer populations with good DNA repair capacity, stable microsatellite instability (MSI-S), mismatch repair proficient (pMMR), low loss of heterozygosity (LOH-L), HRP/BRCA-wt, and TMB <10 mu/mb DNA profiles. Surprisingly, these populations would be expected to contain a high clonal TMB or clonal neoantigen proportion [167]. It is possible that in cancers with low mutational burden, the immune system is challenged to identify clonal neoantigens and requires additional or more durable changes in the immune system, such as a local increase in functional cytokines (i.e., GMCSF) and/or a decrease in immune suppressor cytokine (i.e., TGFβ knockdown) at sites of subclonal and clonal neoantigen education to engage more effective effector cell targeting of the clonal neoantigen. In essence, if tumors are in a low inflammatory microenvironment (cold) as may be expected with low TMB, MSI-S, LOH-L, HRP, BRCA-wt and/or pMMR, establishment of neoantigen targeting experienced effector cells may not be present. If CPI therapy is not administered, the lack of clonally targeting effector cells will deter meaningful CPI-induced clinical benefits. CPI therapy is completely ineffective in anti-cancer activity without sufficient numbers of activated target sensitive effector cells. Such effector cells are likely present to a varying degree in a hot tumor microenvironment; however, they are likely lacking a meaningful presence in a cold tumor microenvironment. The effector cells capable of targeting clonal neoantigen targets are most attractive as they attack the entire cell mass of the malignancy. Transfer of cold to hot tumor microenvironment has been well described as an optimal state of CPI activity [168,169,170]. But the hot microenvironment still requires establishment and presence of clonal targeting effector cells for optimal clinical benefit.

## 7. Limitations of CPI Based Therapy in Newly Diagnosed Advanced Ovarian Cancer

Two Phase 3 studies evaluating CPI in ovarian cancer have failed to show evidence of clinical benefit [171,172]. As a result, there are concerns regarding the further development of immunotherapeutics for ovarian cancer.

In an initial trial of CPIs utility in ovarian cancer, Javelin 100 evaluated either single agent avelumab (*n* = 332) or in combination with chemotherapy (*n* = 331) vs. a control arm of chemotherapy alone (*n* = 335) as frontline maintenance in Stage III-IV ovarian cancer patients following surgical debulking and platinum doublet chemotherapy. The primary endpoint of the trial was PFS. The trial was stopped by the data monitoring committee for futility as the PFS endpoint involving both avelumab cohorts performed worse (single agent; HR 1.43 95% CI 1.05–1.95 *p* = 0.99 and combination HR 1.14 95% CI 0.83–1.56 *p* = 0.79). The median PFS in the avelumab-only group was 16.8 months (95% CI 13.4-not estimable (NE) vs 18.1 months (95% 14.8-NE) in combination therapy and NE (95% 18.2-NE) in the control group [173]. In a subsequent biomarker subset analysis, PD-L1, *BRCA1/2* mutation status and CD8 positivity were not predictive of response to avelumab as a single agent or in combination [174]. The authors concluded that new biomarkers are needed to select appropriate candidates.

Next, in the IMAGYN050 trial (*n* = 1300), atezolizumab was added to frontline chemotherapy and bevacizumab following either primary cytoreductive surgery or interval cytoreduction in patients with newly diagnosed Stage III/IV ovarian cancer. The primary endpoint was PFS. Patients were assigned to either receive atezolizumab with carboplatin plus paclitaxel (CP) and bevacizumab or placebo with CP and bevacizumab. The median PFS was 19.5 months in the atezolizumab containing arm vs. 18.4 months in the placebo arm (HR 0.92 95% CI, 0.79 to 1.07 *p* = 0.28). In a planned exploratory analysis of PD-L1+ patients, there was still no difference in PFS between arms, 20.8 months vs. 18.5 months, (HR 0.80 95% CI, 0.65 to 0.99 *p* = 0.038) [175]. OS data was immature at the time of publication. In either trial, with either single agent CPI or in combination with chemotherapy and bevacizumab, there were no new safety signals identified.

Of note, less than 25% of patients demonstrated >5% PD-L1+ immune cells [175]. In contrast, in tumors known to be immune-responsive, such as non-small cell lung cancer, the PD-L1 expression ranged from 24% to 60% [176]. The low response rate to PD-L1 inhibition in OC could be partially explained by the low expression level of PD-L1 in tumor cells. Additionally, while some studies have demonstrated clinical response in tumors with high TMB, it is notable that ovarian cancer tumors typically demonstrate low TMB [42,177,178,179,180,181,182].

Despite ovarian cancer showing very little responsiveness to CPI therapy, most OC tumors demonstrate the presence of tumor-infiltrating lymphocytes (TILs) with a degree of TIL infiltration that is strongly and reproducibly correlated with survival advantage in other tumor types [183,184]. A meta-analysis of 21 studies and almost 3000 ovarian cancer patients confirmed that high levels of intra-epithelial CD3+ or CD8+ T cells were most significantly associated with both improved progression-free and overall survival [185]. We hypothesize that there may be a threshold of minimum number of clonal neoantigen effector cells available, active and containing clonal TCR sensitivity to engage malignant cells with high clonal and low subclonal neoantigen burden. Technology with capacity to increase the population of effector cells targeting a clonal neoantigen would thus be an obvious course of action and likely attractive combination therapy with CPI. There is evidence that an autologous triple function immunotherapy product, Vigil^®^, may provide such activity [44,45,186,187].

The proportion of tumors containing HRP versus BRCA-m/HRD tumor molecular profile includes nearly 50% of the ovarian cancer population. Clonal TMB or neoantigen levels would be expected to be independent of low numbers of PD-L1+ expressive immune cells [171] and low TMB [179]. Indeed, as we previously described, clonal TMB is a very limited proportion of total TMB. The proportion of HRP profile tumors in ovarian cancer is among the lowest compared to other solid tumor malignancies [188]. For instance, over 80% of bladder cancers, cervical cancers, and prostate cancers have an HRP profile [188]. As discussed below, data for Vigil^®^ support increased clinical benefit in newly diagnosed IIIB-IV BRCA-wt HRP ovarian cancer when Vigil^®^ is used as maintenance therapy following surgical debulking and induction platinum/taxane chemotherapy [44,45,46].

## 8. Vigil^®^—“Full Spectrum” Neoantigen Immune Training in a Permissive Environment

Vigil^®^ is a novel late stage immunotherapy [44,45,46] utilizing GMCSF-wt-bi-shRNA-furin plasmid transfected autologous tumor tissue designed to deliver personalized clonal neoantigen targets, thereby expanding CD8+ T cell clonal neoantigen-targeting effector cells within the tumor microenvironment. In essence, Vigil^®^ is designed to turn “cold” to “hot” within the tumor microenvironment. By introducing immunostimulatory GMCSF and reducing immunosuppressive TGFβ1 and β2 at the intradermal site of inoculation, Vigil^®^ enhances the dendritic and T cell response with the purpose of providing a natural full display of clonal neoantigens. Most, if not all, autologous tumor tissues contain robust clonal neoantigen expression activity at initial presentation, which correlates with effective TCR-responding effector cells to these neoantigens. Specifically, attraction of dendritic cells to the clonal neoantigen expressed on the Vigil tissue following therapeutic intradermal injection enables direct dendritic cell interaction [189]. Dendritic cells are a group of specialized antigen-presenting cells with a key role in the initiation and regulation of innate and adaptive immune response. This interaction is facilitated by Vigil utilizing exposed clonal neoantigens from the autologous tissue used to construct Vigil. Vigil plasmid induces enhancement of local immune function through expression of GMCSF and immune suppression by reduction of TGFβ via activity of bi-shRNA-furin local knockdown. Following the dendritic cell activation targeting a personal clonal neoantigen, dendritic cells move to lymph node tissue to interact with T and B cells and induce a systemic adaptive immune response, thereby expanding immune effector cells that are now able to target the same clonal neoantigen, creating an anti-cancer activity against systemic cancer sites. Evidence of this phenomenon is supported by positive ELISpot assay results across multiple clinical trials where the patient’s mononuclear cells are tested for immunologic response against the patient’s own tumor tissue following Vigil^®^ treatment [190,191,192]. Specifically, Phase 1 testing revealed a correlation between on treatment survival and enzyme-linked immunosorbent spot (ELISpot) response using autologous tumor as the response induction signal against circulating autologous mononuclear cells harvested before and after Vigil^®^ treatment. A positive ELISpot response following therapy was associated with improved OS compared to patients treated by Vigil^®^ who did not demonstrate a ELISpot response (HR 0.23, *p* = 0.038) [190]. ELISpot response appeared to have been tempered in activity in some patients in whom extensive chemotherapy was administered prior to Vigil^®^ therapy, leading to a reduced or no ELISpot response. Follow up Phase 2 testing in patients who had newly diagnosed ovarian cancer and minimal prior chemotherapy to induce immune suppression revealed more impressive ELISpot responses. In newly diagnosed ovarian cancer, 31 of 31 patients who received Vigil^®^ had a positive ELISpot response compared to 0/8 control patients who did not receive Vigil^®^. The ELISpot response at baseline, prior to receiving Vigil^®^, was negative in virtually all patients (30/31). Positive ELISpot results were correlated with improved RFS for Vigil^®^ treated patients compared to similar concurrent control patients who did not receive Vigil^®^ and in whom RFS was not improved (*p* = 0.0165) [192]. Clinical survival benefits have also shown to be durable with long-term follow up beyond 3 years [193]. Moreover, it was shown in original control patients who experienced ovarian cancer relapse and received Vigil^®^ after relapse (*n* = 8), autologous tumor tissue provoked an ELISpot response that was positive in all patients, although the overall level of ELISpot response activity was lower than in patients who received Vigil^®^ as part of frontline maintenance therapy. Whether survival benefit was observed in late Vigil^®^ treatment could not be conclusively determined, although the control patients who crossed over to Vigil^®^ after cancer recurrence were the longest survivors in the control group [193].

**Figure 4:**
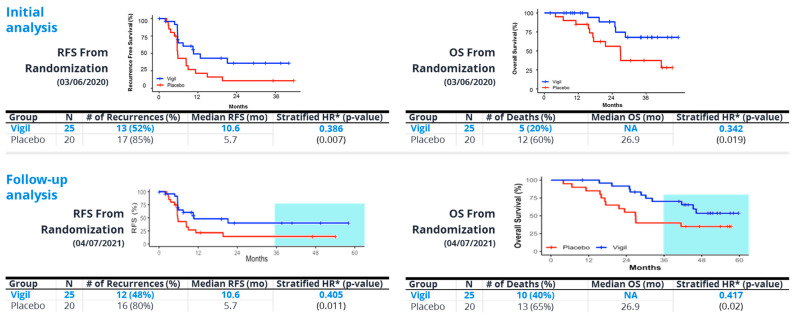
Phase 2b demonstrated statistically significant recurrence-free survival (RFS) and overall survival (OS) results in homologous recombination proficient (HRP) patients compared to placebo; robust differences maintained with further follow up. # = number.

**Figure 5:**
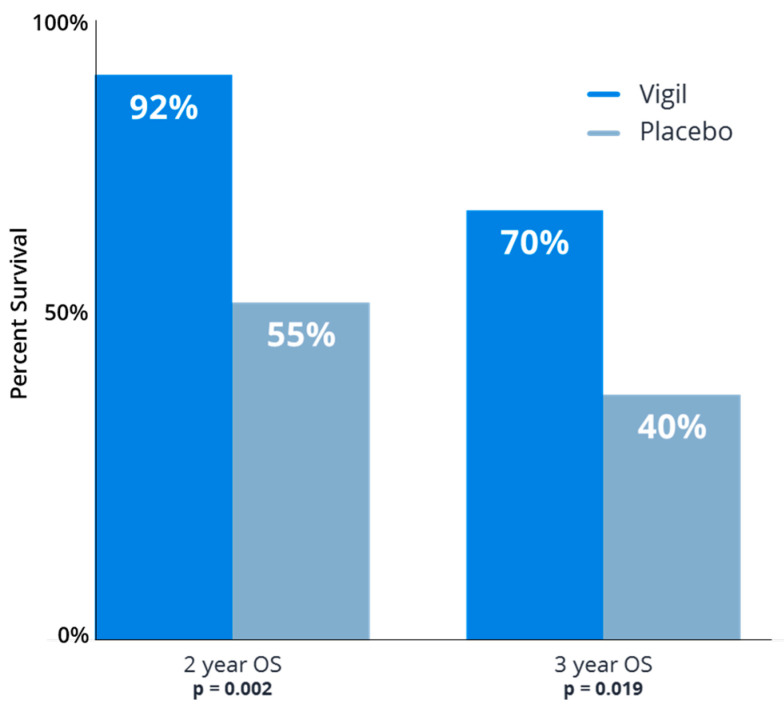
Vigil^®^ improved overall survival following frontline chemotherapy in homologous recombination proficient (HRP) ovarian cancer patients as maintenance therapy. Comparison of Vigil^®^ vs. placebo overall survival (OS) at 2 year and 3 year follow up as maintenance in newly diagnosed Stage IIIb-IV ovarian cancer.

Later, in a double blind Phase 2b trial (VITAL) involving 91 newly diagnosed ovarian cancer patients with Stage IIIb/IV, the results revealed a trend towards benefit from frontline Vigil^®^ maintenance therapy in all patients (HR 0.688, *p* = 0.078). However, analysis of results from patients with maintained tumor DNA repair (i.e., BRCA-wt, HRP) versus impaired DNA repair (BRCA-m, HRD) revealed significant benefit related to Vigil^®^ in both BRCA-wt patients (RFS HR 0.54, *p* = 0.020; OS HR 0.493, *p* = 0.049) and HRP patients (RFS HR 0.386, *p* = 0.007 and OS HR 0.342, *p* = 0.019) [44,45,46]. Kaplan–Meier results are shown in Figure 4. Follow up also revealed that Vigil^®^-treated patients with tumors that had an HRP-positive profile also demonstrated continued sustained benefits in RFS, OS beyond 3 years (Figure 5). These results support the prior assessment that the optimal Vigil^®^-induced response is observed in the HRP-positive population, which relates to good DNA repair and, importantly, a likely higher proportion of clonal neoantigen-expressive tumors [43]. Further work is underway to determine the threshold clonal TMB and tumor-specific clonal neoantigen profiles in Vigil^®^-treated patients to confirm that the observed survival benefit in HRP profile tumors is indeed related to TMB and clonal neoantigen, as hypothesized. Additionally, the relationship between the dendritic cell response and the presentation of clonal neoantigen peptides derived from the tumor cell surface and carried on the dendritic cell MHC-I complex will be explored.

## 9. Conclusions

The sustained clinical benefit of immunoncology therapies has been correlated with the identification of CD8+ T cells recognizing clonal rather than subclonal neoantigens [23,26,42,68,160]. The results presented in this review suggest that a mechanistic approach to identification and targeting clonal tumor mutation burden/neoantigens is potentially the most ideal clinical direction for therapeutic biomarker development to enhance more consistent, effective, and broader CPI utilization. There is a strong suggestion that a wide range of solid tumors may be a target opportunity for CPI treatment based on the assessment and identification of appropriate clonal neoantigens. It is also likely that ovarian cancer is not an immune refractory malignancy, but that the ovarian cancer paradigm must be aligned to subpopulations of patients who have presence of high clonal TMB with accompanying ability to generate clonal targeting effector cells. Moreover, methods to increase presence of clonal neoantigen-targeting effector cells (i.e., Vigil^®^) should be explored. BRCA m and HRD tumors have DNA repair defects which lead to increased mutational load and proportion of subclonal over clonal cancer cells in relationship to HRP, BRCA-wt tumors [23,156,194,195]. The increased presence of subclonal neoantigens will decrease efficiency of clonal effector cell targeting and will also limit the CPI response. As described, tumor clonal TMB and high proportions of clonal neoantigen correlates with OS and PFS improvement to immune therapy, most notably CPI therapy [23,42,68]. The increased subclonal neoantigen heterogeneity in HRD tumors may indicate less effective immunoediting and/or lower expansion of antigen specific CD8+ T cells responses to clonal neoantigens which are present on all the tumor cells. In this scenario, the high clonal neoantigen fraction and clonal TMB in the HRP group could be induced to immune reactivity by Vigil^®^, which creates a permissive immunologic “training” environment for dendritic cells in the skin by presenting the entire antigen repertoire of the patient’s tumor in the presence of immunostimulatory GMCSF and reduced immunosuppressive TGFβ. In essence, Vigil^®^ therapy would create a “hot” tumor microenvironment more supportive of CPI sensitivity. In fact, p53 mutant DNA [196] and ENTPD1 high RNA expression [197] have been identified as relevant immune response biomarkers to Vigil^®^ sensitivity and may further contribute to the induction of a “hot” tumor microenvironment and greater immune responsiveness to the clonal neoantigens that contain the appropriate epitopes for TCR binding and enabling a robust anti-cancer immune response. As quoted by Riaz et al. [25], “even with the brakes off (CPI treatment), the adaptive immune system still must recognize a majority portion (or ideally all) of cancer as foreign in order to facilitate selective elimination of the cancer”. The lack of sufficient presence of clonal neoantigen-targeting effector cells will not provide sufficient capacity of the immune system to enable the CPI anti-cancer effect. Exploration of clonal TMB or neoantigen as a surrogate to clonal neoantigen-targeting effector cells may provide avenues to enhance CPI activity in ovarian cancer and other solid tumor populations that are not as responsive to CPI therapy. Other approaches attempting to impact the targeted anti-tumor immune response (i.e., tumor infiltrating lymphocyte therapy, CAR-T, neoantigen vaccines) would also be expected to benefit from involving clonal neoantigen targeting and expression assessment.

## Figures and Tables

**Table 1:** Summary of treatment responses to FDA-approved checkpoint inhibitors (CPIs) and respective indications. Overall survival (OS); progression free survival (PFS); recurrent free survival (RFS); overall response rate (ORR); microsatellite instability (MSI); mismatch repair deficient (dMMR); duration of response (DoR).

CPI (Target)	Indication/Population	Study (Arms)	Response	Ref.
Pembrolizumab (anti-PD-1)	Unresectable or metastatic BRAF^V600E^ mutated metastatic melanoma, refractory to CTLA-4 therapy and BRAF inhibitor	NCT01295827 (Pembrolizumab)	ORR: 24%	[104]
	Unresectable or metastatic, untreated melanoma regardless or BRAF status	Keynote-006 (Pembrolizumab vs. Ipilimumab)	1-year OS: 74.1% (vs. 58.2%; *p* = 0.0005); 1-year PFS: 47.3% (vs. 26.5%; *p* < 0.001); PD-L1 ≥1%: 24 months OS and PFS: 58% and 33% vs. 45% and 13%, respectively	[105,106]
	Stage III melanoma/adjuvant	Keynote-054 (pembrolizumab vs. placebo)	RFS: 65.33% vs. 49.4%; *p* < 0.0001	[107]
	Previously treated, PD-L1 positive, advanced NSCLC	Keynote-010 (pembrolizumab vs. docetaxel)	OS: 10.4 vs. 8.5 months; *p* = 0.0008	[12,108]
	Untreated, PD-L1 ≥50 advanced NSCLC	Keynote-024 (pembrolizumab vs. chemotherapy)	6 months OS: 80.2% vs. 72.4%; *p* = 0.005 Median PFS: 10.3 vs. 6.0 months; *p* < 0.001 ORR: 44.8% vs. 27.8%	[109]
	Untreated, non-squamous NSCLC without sensitizing mutations, regardless of PD-L1 level	Keynote-021 (pembrolizumab plus chemotherapy vs. chemotherapy	Median PFS: 19.0 vs. 8.9 months (HR: 0.53; *p* = 0.0049) ORR: 56.7% vs. 26.4%; *p* = 0.0016 Note: data from Keynote-189 and 407 revealed consistent PFS and OS advantage for pembrolizumab	[110,111,112]
	Recurrent/metastatic head and neck squamous cancer; 2nd line or 1st line in CPS ≥1	Keynote-012 (pembrolizumab)	ORR: 16%	[113,114]
	Unresectable/metastatic urothelial cancer; 2nd line	Keynote-045 (pembrolizumab)	Median OS: 10.3 vs. 7.4 months (*p* = 0.002)	[115]
	Unresectable/metastatic urothelial cancer, cisplatin-ineligible; 1st line in CPS ≥10	Keynote-052 (pembrolizumab)	ORR: 38%	[116]
	MSI-H, dMMR or TMB-H solid tumors	Keynote-012, 016, 028, 158 * and 164 (pembrolizumab)	MSI-H or dMMR studies: ORRs ~ 40% TMB-H: ORR: 29% (vs. 6% TMB-L) Cervical cancer cohort/MSI-H or dMMR: ORR: 12.2% Endometrial cancer cohort/MSI-H or dMMR: ORR: 48%	[117,118,119]
	Persistent/recurrent or metastatic cervical cancer with PD-L1 CPS ≥ 1	Keynote-826 (pembrolizumab vs. placebo + paclitaxel/platinum +/− bevacizumab	Median OS: not reached vs. 16.3 months (*p* = 0.0001) Median PFS: 10.4 vs. 8.2 months (*p* < 0.0001) ORR: 68% vs. 50%; median DOR: 18 vs. 10.4 months	[120]
Nivolumab (anti-PD-1)	Persistent/recurrent cervical cancer	NRG-GY002 (nivolumab)	6 months PFS: 16%; 6 months OS: 78% Median duration of SD: 5.7 months	[121]
	Persistent/recurrent cervical cancer	CheckMate-358 (nivolumab)	ORR: 26.3% Median OS: 21.9 months	[122]
Cemiplimab (anti-PD-1)	Recurrent or metastatic cervical cancer after platinum-based chemotherapy, regardless of PD-L1	NCT03257267 (cemiplimab vs. investigator-choice chemotherapy)	ORR: 16.4% vs. 6.3% Median OS: 12.0 vs. 8.5 months (*p* < 0.001) Median PFS: HR 0.75, *p* < 0.001	[123]
Dostarlimab-gxly (anti-PD-1)	Primary advanced or recurrent dMMR or MSI-H endometrial cancer	RUBY (dostarlimab-gxly vs. placebo + carboplatin-paclitaxel, followed by dostarlimab or placebo	Median PFS: 30.3 vs. 7.7 months (*p* < 0.0001)	[124]
Avelumab (anti-PD-L1)	Chemo-refractory, metastatic Merkel cell carcinoma, regardless of PD-L1	NCT02155647 (Avelumab)	ORR: 31.8% (28/88); CR: 8/88 and PR: 20/88	[125]
	Advanced urothelial carcinoma, 2nd line setting	JAVELIN Solid Tumor (Avelumab)	ORR: 16.5%; CR: 4.1%; PR: 12.4% Median DoR: 20.5 months Median PFS: 1.6 months Median OS: 7.0 months; 24-month OS: 20.1%	[126,127]
	Advanced renal cell carcinoma (1st line in combination with Axitinib	JAVELIN Renal (Avelumab + Axitinib vs. sunitinib)	PD-L1+: median PFS: 13.8 vs. 7.2 months (*p* < 0.001); ORR: 55.2% (vs. 25.5%) Overall population: median PFS: 13.8 vs. 8.4 (*p* < 0.001)	[128]
Durvalumab (anti-PD-L1)	Advanced/metastatic urothelial carcinoma, platinum-refractory	NCT01693562 (Durvalumab)	ORR: 31% (all patients), 46.4% (PD-L1 positive), 0% (PD-L1 negative)	[129]
	Stage III NSCLC, maintenance therapy, post-chemo-radiation	PACIFIC Trial (Durvalumab vs. placebo)	Median PFS: 16.8 vs. 5.6 months (*p* < 0.001) ORR: 28.4% vs. 16% (*p* < 0.001)	[130]
Atezolizumab (anti-PD-L1)	Metastatic urothelial carcinoma, platinum ineligible or refractory, regardless of PD-L1	IMvigor210 (Atezolizumab)	ORR: 23% Median PFS: 2.7 months Median OS: 15.9 months	[131]
OK	Metastatic NSCLC, 2nd line post platinum-based chemotherapy, regardless of PD-L1	OAK/POPLAR (Atezolizumab vs. docetaxel)	Median OS: 12.6–13.3 vs. 9.7–9.8 months (*p* = 0.05)	[132,133]
	Extensive stage SCLC, 1st line setting	IMpower 133 (Chemo/atezolizumab vs. chemotherapy/ placebo)	Median OS: 12.3 vs. 10.3 months (*p* = 0.007) Median PFS: 5.2 vs. 4.3 months (*p* = 0.02)	[134]

* Included 98 patients with advanced cervical cancer (ORR: 12.2%; 12/98) and 90 patients with advanced endometrial cancer (ORR: 48%; 43/90).

**Table 2:** Human syndromes with defective genome maintenance.

Syndrome	Affected Maintenance Mechanism	Main Type of Genome Instability	Major Cancer Predisposition
Xeroderma pigmentosum	NER (±Transcription coupled repair)	Point mutations	UV-induced skin cancer
Cockayne syndrome	Transcription coupled repair	Point mutations	None *
Trichothiodystrophy	NER/Transcription coupled repair	Point mutations	None *
Ataxia telangiectasia (AT)	DSB response/repair	Chromosome aberrations	Lymphomas
AT-like disorder	DSB response/repair	Chromosome aberrations	Lymphomas
Nijmegen breakage syndrome	DSB response/repair	Chromosome aberrations	Lymphomas
BRCA 1/BRCA 2	Homologous recombination	Chromosome aberrations	Breast (ovarian) cancer
Werner syndrome	Homologous recombination/TLS	Chromosome aberrations	Various cancers
Bloom syndrome	Homologous recombination	Chromosome aberrations (SCE↑)	Leukemia, lymphoma, others
Rothmund-Thomson syndrome	Homologous recombination	Chromosome aberrations	Osteosarcoma
Ligase IV deficiency ^†^	EJ	Recombination fidelity	Leukemia
HNPCC	MMR	Point mutations	Colorectal cancer
Xeroderma pigmentosum variant	TLS ^‡^	Point mutations	UV-induced skin cancer
ERCC6L2 deficiency	NER	Point mutations	hematologic
Constitutional mismatch repair disorder	MMR	Point mutations and insertion/deletions	Hematologic, brain and intestinal tract
Fanconi Anemia	FA	Chromosomal aberrations	SCC, AML, MDS

* Defect in transcription-coupled repair triggers apoptosis, which may protect against UV-induced cancer. ^†^ One patient with leukemia and radiosensitivity described with active-site mutation in ligase IV. ^‡^ Specific defect in relatively error-free bypass replication of UV-induced cyclobutene pyrimidine dimers. Abbreviations: base-excision repair (BER); double-strand break (DSB); hereditary non-polyposis colorectal cancer (HNPCC); mismatch repair (MMR); nucleotide-excision repair (NER); sister-chromatid exchange (SCE); translesion synthesis (TLS); mismatch repair (MMR); nucleotide excision repair (NER); end joining (EJ); squamous cell carcinoma (SCC); acute myeloid leukemia (AML); myelodysplastic syndrome (MDS); Fanconi anemia (FA).

## Data Availability

Data are contained within the article.
